# Long-Term Retinal Neurovascular and Choroidal Changes After Panretinal Photocoagulation in Diabetic Retinopathy

**DOI:** 10.3389/fmed.2021.752538

**Published:** 2021-10-18

**Authors:** Tian Huang, Xiaoli Li, Jie Xie, Liang Zhang, Guanrong Zhang, Aiping Zhang, Xiangting Chen, Ying Cui, Qianli Meng

**Affiliations:** ^1^Department of Ophthalmology, Guangdong Provincial People's Hospital, Guangdong Eye Institute, The Second School of Clinical Medicine, Southern Medical University, Guangdong Academy of Medical Sciences, Guangzhou, China; ^2^State Key Laboratory of Ophthalmology, Zhongshan Ophthalmic Center, Sun Yat-sen University, Guangzhou, China; ^3^Information and Statistical Center, Guangdong Provincial People's Hospital, Guangdong Academy of Medical Sciences, Guangzhou, China; ^4^Department of Ophthalmology, Guangzhou First People's Hospital, Guangzhou, China

**Keywords:** choroid, diabetic retinopathy, microvasculature, neural retina, optical coherence tomography angiography, panretinal photocoagulation

## Abstract

**Purpose:** To evaluate the long-term retinal microvascular, neural, and choroidal changes in the patients with severe nonproliferative diabetic retinopathy (NPDR) and proliferative diabetic retinopathy (PDR) following panretinal photocoagulation (PRP).

**Methods:** Forty-five eyes of 28 patients with treatment-naive severe NPDR and PDR were included and followed for 12 months after PRP. Microvascular and neural changes in the macular and peripapillary areas were assessed by using optical coherence tomography angiography. Subfoveal choroidal thickness (SFCT) was measured by using optical coherence tomography. A Linear mixed-effects model was used to highlight the differences for the variables after adjusting for sex, age, and axial length.

**Results:** Compared to baseline, there were no statistical differences in the best corrected visual acuity (BCVA), macular and peripapillary vessel density (VD), and SFCT following PRP. Macular thickness significantly increased at 1 and 3–6 months after PRP (*p* < 0.05), while the peripapillary retinal nerve fiber layer (RNFL) and ganglion cell complex (GCC) thickness significantly increased at 1 month postoperatively (*p* < 0.01). Global loss volume and focal loss volume significantly decreased at the same time point (*p* < 0.05).

**Conclusion:** The unchanged BCVA, VD, the thickness of RNFL and GCC, and SFCT during the 12-month follow-up period suggest that PRP may prevent the retinal neurovascular and choroidal damage.

## Introduction

Diabetic retinopathy (DR) is one of the most common microvascular complications of diabetes and is the leading cause of visual impairment among the working-age population across the globe (1). Changes in the retinal microvascular and neural structure due to diabetes are considered fundamental to the pathophysiology and progression of DR. Reduction of the choroidal thickness (CT) (2, 3) and choroidal perfusion (4, 5) has also been reported in DR. Panretinal photocoagulation (PRP) is demonstrated to prevent the impairment of visual function due to the proliferative diabetic retinopathy (PDR) and diabetic macular edema (DME) in the landmark clinical trials such as the Diabetic Retinopathy Study (DRS) (6, 7) and the Early Treatment Diabetic Retinopathy Study (ETDRS) (8). The physiological mechanism of PRP is generally considered to involve the physical light energy destroying the photoreceptors and decreasing oxygen consumption, which improves oxygen flux to reach the inner retina, relieves inner retinal hypoxia, and raises the oxygen tension, resulting in reducing or stopping the production of the growth factors and neovascularization (9, 10).

Since fundus fluorescein angiography (FFA) is a gold standard for the diagnosis and grading of DR, the neural and choroidal changes cannot be evaluated by FFA with two-dimensional images. Emerging as a noninvasive and depth-resolved imaging technique without dye injection, optical coherence tomography angiography (OCTA) allows for fast visualization of the macular microvasculature, differentiation of various retinal vascular layers, and quantification of vessel density or thickness of macular area, retinal nerve fiber layer (RNFL), and ganglion cell complex (GCC). Accumulating evidence supports OCTA as a potential tool for the evaluation of the severity of DR and efficacy of the therapies for DR (11, 12). A previous study quantified a set of the neurovascular parameters of OCTA related to the severity of DR, which might have potential clinical applications for DR staging (11). Previous longitudinal studies have shown that the microangiopathy and neurodegeneration appear in parallel and are highly progressive even in the early stage of DR (13), but few studies explored these features following PRP. To the best of our knowledge, the comprehensive retinal vascular and neural changes in the macular and peripapillary area and subfoveal choroidal thickness (SFCT) changes after PRP have not been reported.

In this study, we present a prospective, longitudinal, and observational study to investigate the retinal microvascular, neural, and choroidal changes up to 1 year after PRP in severe non-PDR (NPDR) and PDR by using multimodal imaging.

## Materials and Methods

This study was conducted at the Department of Ophthalmology of Guangdong Provincial People's Hospital between October 2018 and June 2020. All the procedures adhered to the tenets of the Declaration of Helsinki and were approved by the research ethics committee of Guangdong Provincial People's Hospital. All the participants provided written informed consent.

### Patients

**Figure 1:**
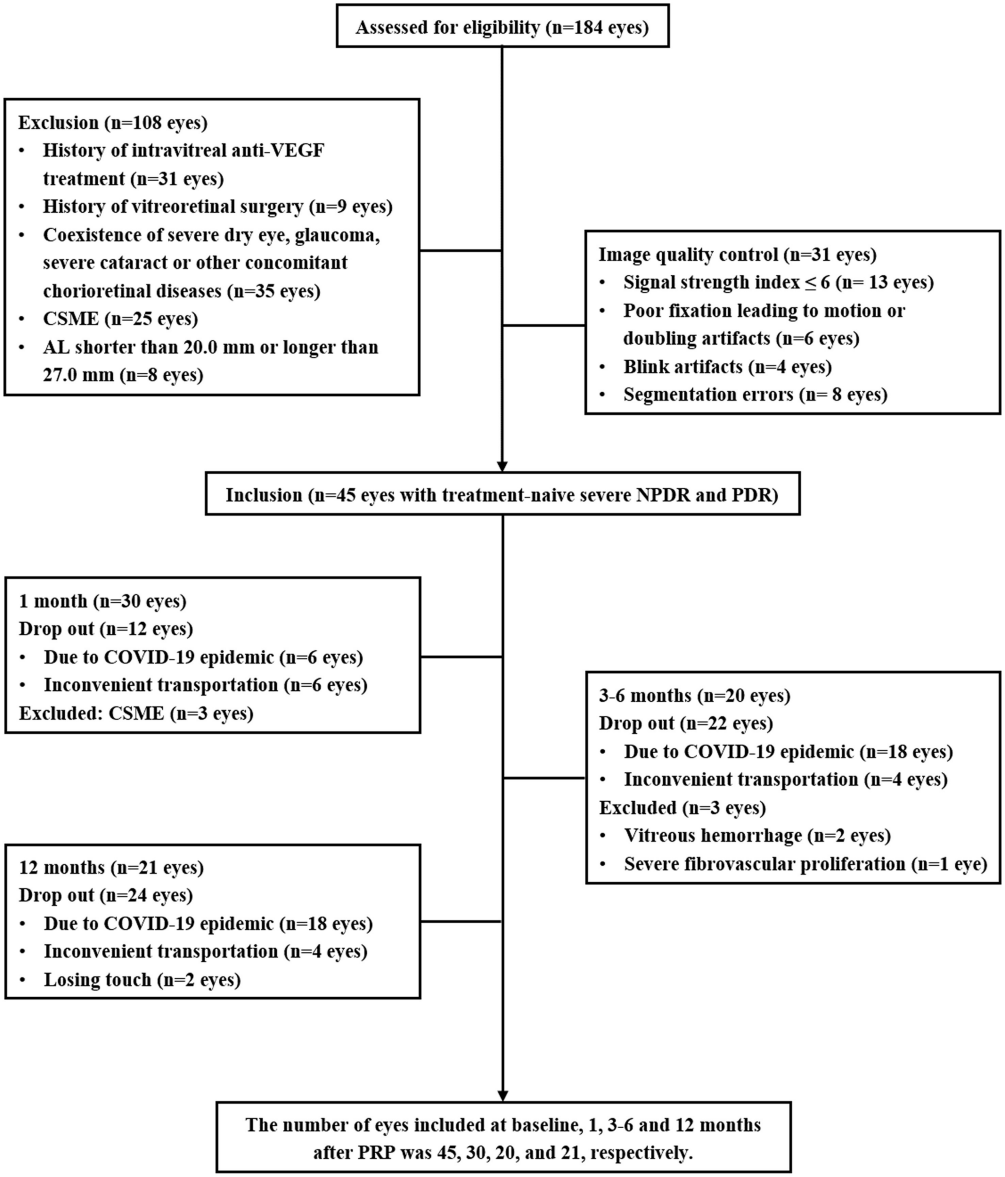
Flowchart detailing enrollment and follow-up of the subjects in the study.

Patients with type 2 diabetic diagnosed with severe NPDR or PDR who were the candidates for PRP treatment were enrolled. The DR assessment and grading were based on the FFA and color fundus photography (CFP) by using the proposed international Diabetic Retinopathy Severity Scale (14). The exclusion criteria, as shown in Figure 1, included the history of ophthalmic intervention, the coexistence of the other ocular diseases affecting the assessment, clinically significant macular edema (CSME) (central subfield macular thickness ≥ 300 μm) (15), axial length (AL) <20.0 mm or > 27.0 mm, and the low-quality OCTA images.

### Ophthalmic and Systemic Examinations

A comprehensive ophthalmic examination was performed in all the participants at baseline and at 1, 3–6, and 12 months after PRP including best corrected visual acuity (BCVA), intraocular pressure, slit-lamp examination, dilated fundoscopy, 45° CFP (TRC-NW8 fundus camera, TOPCON, Tokyo, Japan), OCT (HRA-OCT, Heidelberg Engineering, Germany), and OCTA (Optovue, Fremont, California, USA). FFA (Spectralis HRA, Heidelberg Engineering, Jena, Germany) was performed at baseline and 3–6 and 12 months after PRP. AL was obtained by using ocular biometry (LS900 Haag-Streit Diagnostics, Köniz, Switzerland) at baseline to correct the retinal magnification of each OCTA image.

Demographic and systemic parameters including the age, sex, duration of diabetes, blood pressure, body mass index (BMI), waist-hip ratio (WHR), glycated hemoglobin (HbA1c), creatinine, and creatinine clearance rate (Ccr) within 1 week before initiating PRP were recorded.

### Optical Coherence Tomography Angiography and OCT Image Process

The OCTA images were obtained by using the split-spectrum amplitude-decorrelation angiography algorithm incorporated with the RTVue XR Avanti device with AngioVue 2.0 (Optovue Inc., Fremont, California, USA) (16). In this study, the OCTA image acquisition and parameter analysis are described in detail (11). In brief, HD Angio Retina 6.0 mm scan centered on the fovea was used to assess the retinal vessel density (VD), macular thickness, and foveal avascular zone (FAZ). Macular VD and thickness were distinctly evaluated in both the superficial capillary plexus (SCP) and deep capillary plexus (DCP) within the ETDRS 6 × 6 whole grid. The FAZ parameters, including the FAZ area, FAZ perimeter, acircularity index (AI), and FD-300, were evaluated by using the nonflow area tool to provide the automated FAZ segmentation. HD Angio Disk 4.5 mm scan centered on the optic disk was used to quantify the inside of disk and peripapillary VD and the RNFL thickness in the radial peripapillary capillary segment (refer to figure, Supplementary Figure 1, which shows the angio retina and disk measurement zones). The GCC scan focused on the ganglion cells was used to automatically calculate the GCC thickness, global loss volume (GLV), and focal loss volume (FLV). SFCT was assessed by using OCT with the enhanced depth-imaging (OCT-EDI) technique.

Optical coherence tomography angiography and OCT images were independently evaluated by the two masked ophthalmologists (XL and YC) at different time points and in different orders. A third trained grader (QM) adjudicated all the cases of discrepancy. All the images were evaluated on the instrument display screen in a standardized and dimmed environment. Manual segmentation was performed to correct the automated segmentation errors when needed.

### Panretinal Photocoagulation

All the participants received the first PRP session within 3 days of the baseline evaluation by using a frequency doubled scan laser relying on a 532 nm (green) light according to the recommendations of the ETDRS (6). PRP was performed in the three or four sessions with an interval of 1 week between the sessions. The retinal spot size of 200 μm and laser duration of 0.2 s were employed. The effective power of the laser was determined by the yellowish-white coagulative spots. The average number of the shots was 1,373 burns (range: 868–1,982). Treatment was deemed adequate when no further neovascularization or hemorrhage was detected during follow-up visits at 1, 3–6, and 12 months after the final laser treatment session.

### Statistical Analysis

The statistical analyses were performed by using SPSS version 25.0 software (SPSS Inc., Chicago, Illinois, USA). Graphs were plotted by using GraphPad Prism 8 (GraphPad Software, San Diego, California, USA). Quantitative variables were tested with Shapiro–Wilk to determine the normality and presented as mean ± SD, median (interquartile range), or 95% CI. For statistical analysis, visual acuity was converted to the logarithm of the minimum angle of resolution (LogMAR). Descriptive statistics were used to characterize the demographics of the study population. A linear mixed-effects model was used to highlight the differences for the continuous OCTA and OCT parametric variables, adjusting for sex, age, and AL. All the statistical tests were two-sided and a *p* < 0.05 was considered statistically significant.

## Results

In this prospective study, a total of 45 eyes with treatment-naive severe NPDR and PDR from 28 patients with type 2 diabetes were enrolled. An overview of the demographic and clinical characteristics at baseline and 12 months after PRP is presented in Table 1. To avoid the effects of antivascular endothelial growth factor (VEGF) intravitreal injection (IVI) and vitrectomy on the retinal neurovascular and choroidal parameters measured by OCTA and OCT, the patients who received IVI for CSME or vitrectomy for the vitreous hemorrhage and severe fibrovascular proliferation during the follow-up were excluded. Some participants were lost to follow-up due to the novel coronavirus disease 2019 (COVID-19). Thus, the number of eyes included in 1, 3–6, and 12 months after PRP was 30, 20, and 21, respectively (Figure 1).

**Table 1:** Demographic and systemic characteristics of the participants.

**Characteristics**	**Mean** **±** **SD/Median (interquartile range)**		***P*-value**
	**Baseline**	**12 months after PRP**	
Patients (eyes)	28 (45)	13 (21)	
Sex, male (%)	14 (50%)	8 (62%)	
Duration of diabetes	11.79 ± 8.23	–	
(years)			
Age (years)	57.46 ± 8.67	58.54 ± 8.78	0.772
BMI (kg/m^2^)	23.09 ± 2.50	23.04 ± 2.52	0.943
SBP (mmHg)	139.61 ± 19.63	139.21 ± 19.19	0.940
DBP (mmHg)	79.71 ± 13.40	80.57 ± 13.18	0.810
HbA1c (%)	8.26 ± 2.07	8.07 ± 1.92	0.724
Creatinine (μmol/L)	92.45 (70.16–123.37)	94.25 (71.36–128.54)	0.781
Ccr (ml/min)	61.66 ± 23.78	60.69 ± 24.82	0.776
AL (mm)	22.85 ± 0.63	22.90 ± 0.64	0.455

*p-values are calculated by using a t-test*.

*SBP, systolic blood pressure; DBP, diastolic blood pressure; BMI, body mass index; HbA1c, glycated hemoglobin; Ccr, creatinine clearance rate; AL, axial length*.

There were no significant differences in age, BMI, blood pressure, HbA1c, and renal function at baseline and at 12 months after PRP. The BCVA was LogMAR 0.20 (95% CI 0.10–0.29) at baseline adjusting for the age, sex, and AL and showed an improved trend after PRP, although no statistical difference was observed (Table 2).

**Table 2:** Longitudinal changes of the best corrected visual acuity (BCVA), subfoveal choroidal thickness (SFCT), macular vessel density, and foveal avascular zone (FAZ) after panretinal photocoagulation [mean (95% CI)].

**Variables**	**Baseline (*n* = 45)**	**1 month (*n* = 30)**	**3-6 months (*n* = 20)**	**12 months (*n* = 21)**	***P*-value**
BCVA (LogMAR)	0.20 (0.10–0.29)	0.14 (0.05–0.23)	0.15 (0.07–0.23)	0.14 (0.06–0.23)	0.355
SFCT (μm)	252.43 (220.92–283.95)	259.98 (227.48–292.47)	261.79 (229.11–294.47)	263.73 (230.08–297.39)	0.507
Macular SCP VD (%)					
Whole VD	46.27 (43.88–48.66)	46.45 (44.17–48.73)	45.67 (43.34–48.00)	46.06 (43.66–48.46)	0.593
Foveal VD	17.26 (12.84–21.69)	20.66 (16.01–25.31)	17.52 (13.30–21.74)	18.5 (14.07–22.93)	0.094
Parafoveal VD	45.80 (42.74–48.87)	45.99 (43.13–48.86)	45.03 (42.14–47.92)	44.79 (41.82–47.77)	0.391
Perifoveal VD	47.21 (44.73–49.70)	47.59 (45.21–49.97)	46.79 (44.3–49.28)	46.94 (44.44–49.44)	0.579
Macular DCP VD (%)					
Whole VD	43.05 (40.79–45.31)	42.91 (40.84–44.97)	42.38 (40.01–44.76)	43.65 (41.21–46.09)	0.703
Foveal VD	27.52 (23.28–31.75)	28.17 (23.9–32.43)	27.86 (23.77–31.95)	27.51 (23.11–31.91)	0.942
Parafoveal VD	47.43 (45.27–49.59)	46.89 (44.77–49.01)	46.28 (43.93–48.62)	47.88 (45.56–50.2)	0.443
Perifoveal VD	43.93 (41.50–46.37)	44.10 (41.82–46.38)	43.52 (41.03–46.01)	44.72 (42.08–47.37)	0.746
FAZ (6.0 mm scan)					
FAZ (mm^2^)	0.38 (0.30–0.46)	0.32 (0.23–0.41)	0.34 (0.25–0.44)	0.36 (0.26–0.46)	0.412
Perimeter (mm)	2.49 (2.21–2.77)	2.26 (1.94–2.57)	2.33 (1.99–2.76)	2.41 (2.06–2.76)	0.396
AI	1.16 (1.13–1.20)	1.15 (1.11–1.19)	1.13 (1.09–1.17)	1.16 (1.11–1.20)	0.376
FD-300	47.17 (44.85–49.49)	48.41 (45.84–50.98)	45.22 (42.52–47.92)	45.78 (43.02–48.54)	0.108
Peripapillary VD (%)					
Peripapillary	49.02 (47.32–50.72)	48.12 (46.28–49.97)	48.67 (46.78–50.55)	47.52 (45.59–49.45)	0.146
Superior Nasal	45.90 (43.76–48.04)	44.93 (42.48–47.37)	46.81 (44.32–49.30)	45.09 (42.52–47.67)	0.444
Nasal Superior	43.60 (41.26–45.95)	42.67 (40.15–45.2)	43.76 (41.19–46.32)	42.04 (39.42–44.66)	0.235
Nasal Inferior	43.90 (42.05–45.76)	43.98 (41.84–46.11)	43.17 (40.97–45.37)	43.18 (40.9–45.46)	0.848
Inferior Nasal	48.48 (45.69–51.26)	47.62 (44.63–50.6)	47.72 (44.69–50.74)	46.24 (43.16–49.33)	0.174
Inferior Temporal	55.37 (52.79–57.95)	53.76 (50.92–56.59)	54.51 (51.61–57.40)	54.47 (51.50–57.45)	0.415
Temporal Inferior	51.92 (50.06–53.78)	51.35 (49.21–53.50)	53.16 (50.95–55.38)	51.13 (48.83–53.42)	0.359
Temporal Superior	54.25 (52.01–56.5)	53.79 (51.14–56.45)	53.86 (51.10–56.63)	54.87 (51.99–57.75)	0.896
Superior Temporal	51.92 (49.06–54.78)	51.50 (48.23–54.77)	52.09 (48.72–55.45)	51.96 (48.48–55.44)	0.986

*p-values are calculated by using a linear mixed-effects model before and after panretinal photocoagulation adjusting for age, sex, and axial length*.

*LogMAR, the logarithm of the minimum angle of resolution; SCP, superficial capillary plexus; VD, vessel density; DCP, deep capillary plexus; AI = acircularity index*.

### Longitudinal Changes of the Retinal Vessel Density, Thickness, and FAZ in the Macular Area After PRP

**Figure 2:**
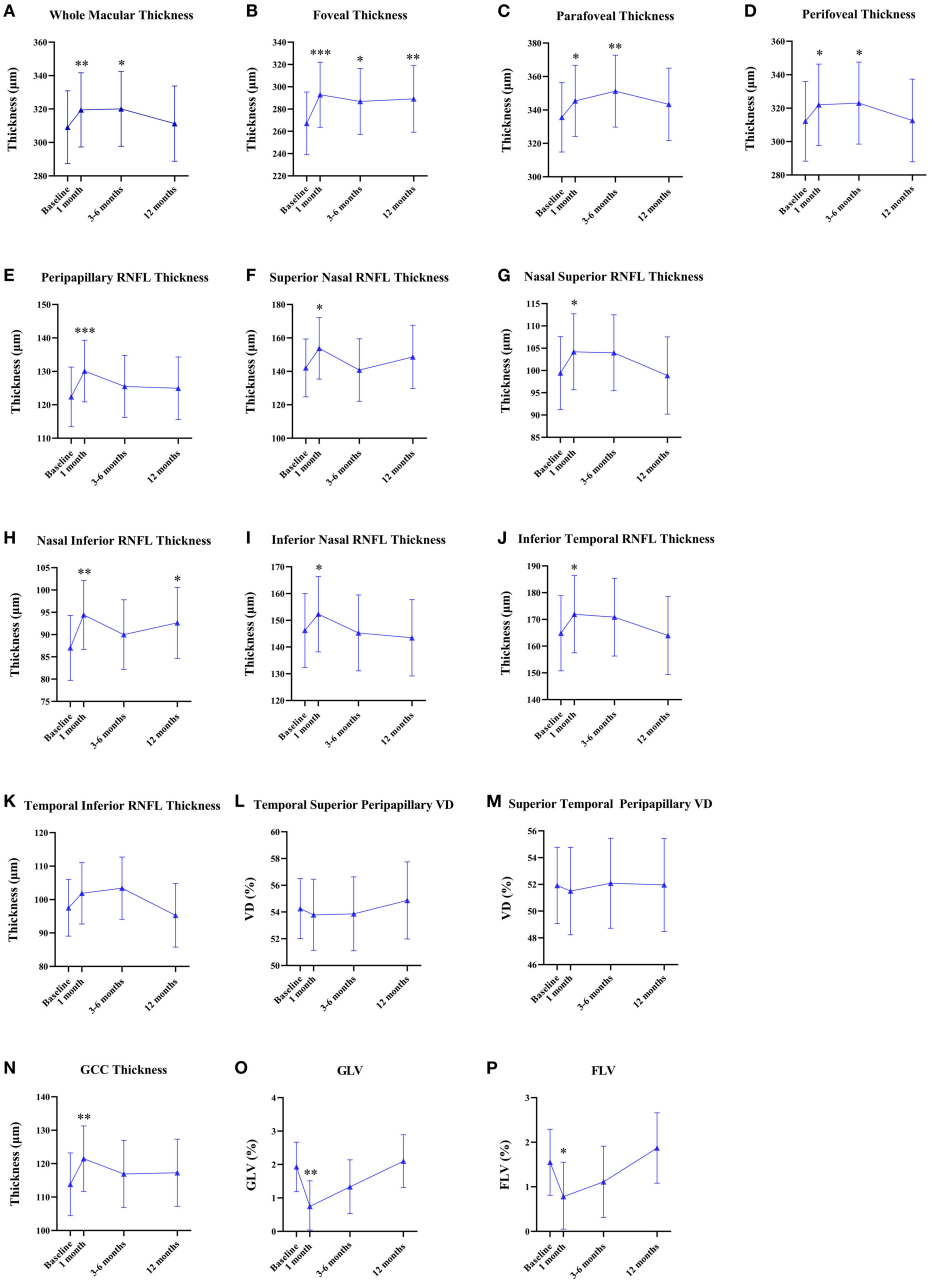
Quantitative analyses of the longitudinal microvascular and neural changes in the macular and peripapillary areas during 12 months after panretinal photocoagulation (PRP). **(A–D)** Longitudinal changes of macular thickness [mean (95% CI), μm] after PRP. **(E–M)** Longitudinal changes of peripapillary retinal nerve fiber layer (RNFL) thickness [mean (95% CI), μm] after PRP. **(N–P)** Longitudinal changes of the ganglion cell complex (GCC) thickness [mean (95% CI), μm]; global loss volume (GLV) [mean (95% CI), %]; and focal loss volume (FLV) [mean (95% CI), %] after PRP. *p < 0.05, **p < 0.01, ***p < 0.001.

There were no statistical differences in the macular VD including SCP and DCP and FAZ between the baseline and after PRP (Table 2), while macular thickness including whole parafoveal areas and perifoveal areas was significantly increased at 1 and 3–6 months (whole macular thickness: *p* = 0.007, *p* = 0.016, respectively; parafoveal thickness: *p* = 0.022, *p* = 0.002, respectively; and perifoveal areas: *p* = 0.023, *p* = 0.034), but then declined at 12 months after PRP. However, increased foveal thickness persisted up to 12 months postoperatively (*p* < 0.001, *p* = 0.019, *p* = 0.007, respectively) (Figures 2A–D, Supplementary Table 1).

### Longitudinal Changes of the Peripapillary VD and RNFL Thickness After PRP

None of the statistical changes were observed in the whole peripapillary VD and eight peripapillary segments (Table 2). Compared to baseline, RNFL thickness in the whole peripapillary area and seven peripapillary segments except for the temporal inferior segment significantly increased at 1 month after PRP (whole peripapillary RNFL thickness, superior nasal peripapillary RNFL thickness, nasal superior peripapillary RNFL thickness, nasal inferior peripapillary RNFL thickness, inferior nasal peripapillary RNFL thickness, inferior temporal peripapillary RNFL thickness, temporal superior peripapillary RNFL thickness, superior temporal peripapillary RNFL thickness: *p* < 0.001, *p* = 0.023, *p* = 0.018, *p* = 0.001, *p* = 0.013, *p* = 0.013, *p* = 0.031, *p* = 0.003, respectively), while temporal inferior RNFL thickness was unchanged during 12 months. However, the nasal inferior RNFL thickness was significantly increased at 1 and 12 months (1 month: *p* = 0.001, 12 months: *p* = 0.025) (Figures 2E–M, Supplementary Table 1).

### Longitudinal Changes of the GCC Thickness, GLV, and FLV After PRP

Figures 2N–P shows the GCC thickness, GLV, and FLV that were stable at 12-month follow-up. However, compared to baseline, GCC thickness significantly increased (*p* = 0.005) and GLV and FLV significantly decreased at 1 month postoperatively (*p* = 0.003, *p* = 0.013, respectively) (Supplemental Table 1).

### Longitudinal Changes of SFCT After PRP

Table 2 shows the mean SFCT that was 252.43 μm (95% CI 220.92–283.95) at baseline. No statistical differences of SFCT were observed during the follow-up visit after PRP, although an increasing trend was shown.

### Longitudinal Morphological Changes of Vascular Abnormality by Using the Multimodal Images

**Figure 3:**
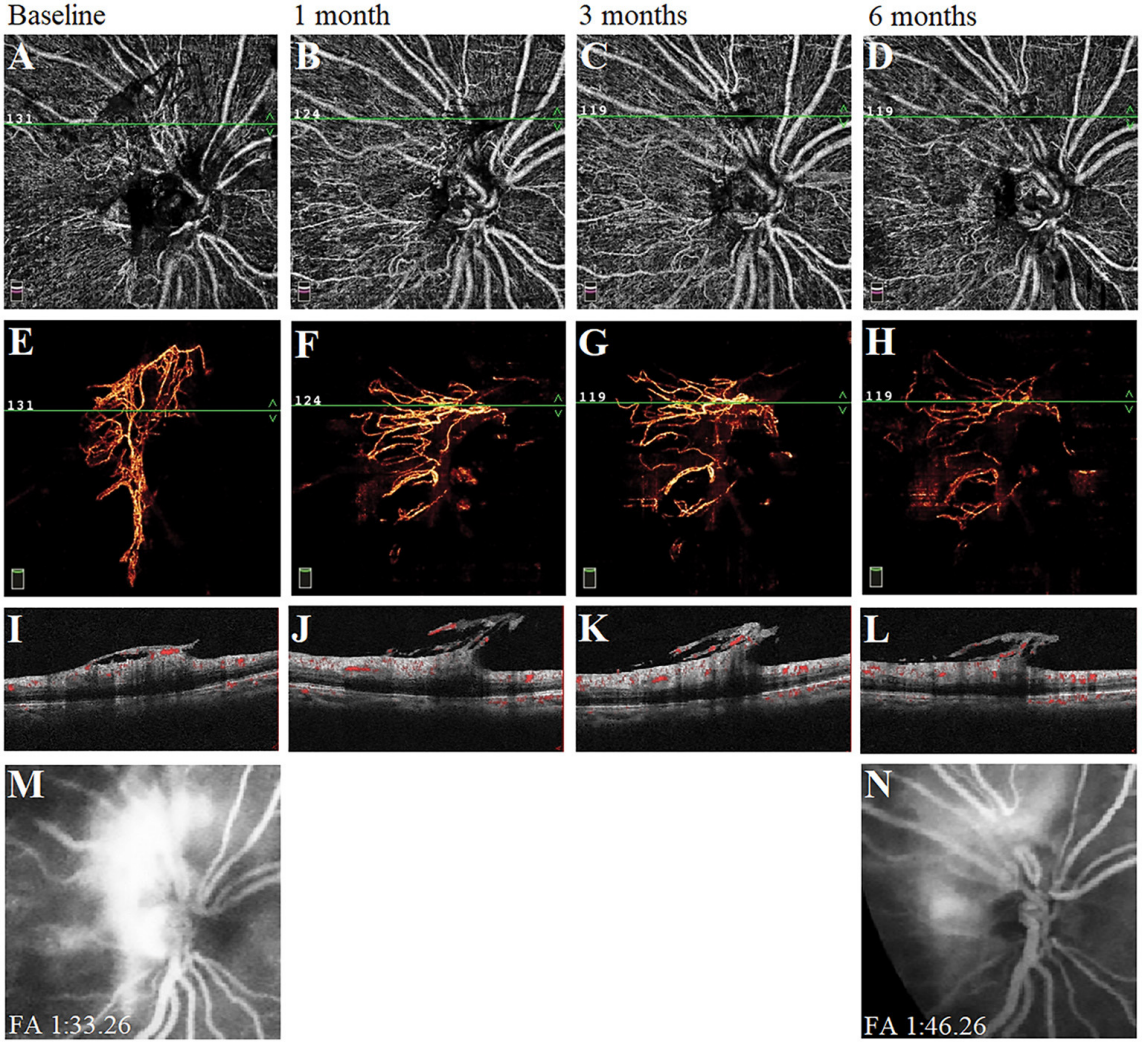
A typical proliferative diabetic retinopathy case showed the neovascularization of the disk (NVD) changed from large, dense, and strong fluorescein leakage to small, loose, and weak fluorescein leakage at 6 months after panretinal photocoagulation (PRP). **(A–D)** Retina en-face slab images of NVD were observed by optical coherence tomography angiography (OCTA). **(E–H)** Vitreous en-face slab images of OCTA. **(I–L)** B-scan image of OCTA. **(M,N)** Images of fluorescein angiography at baseline and 6 months.

**Figure 4:**
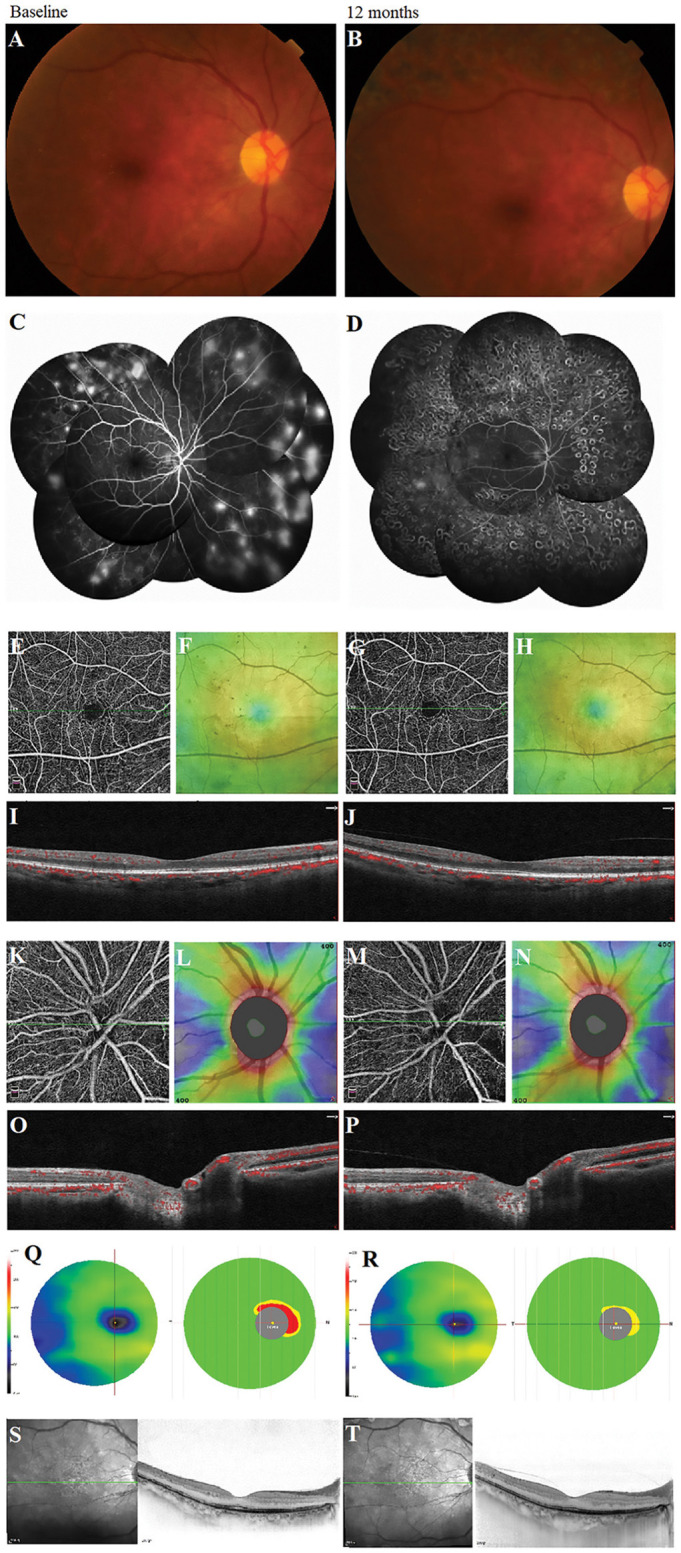
A typical proliferative diabetic retinopathy case showed the retinal neovascularization regression with the unchanged vessel density, the thickness of retinal nerve fiber layer and ganglion cell complex, and subfoveal choroidal thickness at 12 months after panretinal photocoagulation (PRP) in the multimodal images. **(A,B)** Images of color fundus photography. **(C,D)** Images of fundus fluorescein angiography. **(E,G)** Retina en-face slab images in HD Angio Retina 6.0 mm scan by optical coherence tomography angiography (OCTA). **(F,H)** Full retinal thickness images in HD Angio Retina 6.0 mm scan of OCTA. **(I,J)** B-scan images in HD Angio Retina 6.0 mm scan of OCTA. **(K,M)** Retina en-face slab images in HD Angio Disk 4.5 mm scan by OCTA. **(L,N)** Retinal nerve fiber layer thickness images of OCTA. **(O,P)** B-scan images in HD Angio Retina 6.0 mm scan of OCTA. **(Q,R)** Ganglion cell complex scan images of OCTA. **(S,T)** Images of the OCT with the enhanced depth-imaging (OCT-EDI) technique.

**Figure 5:**
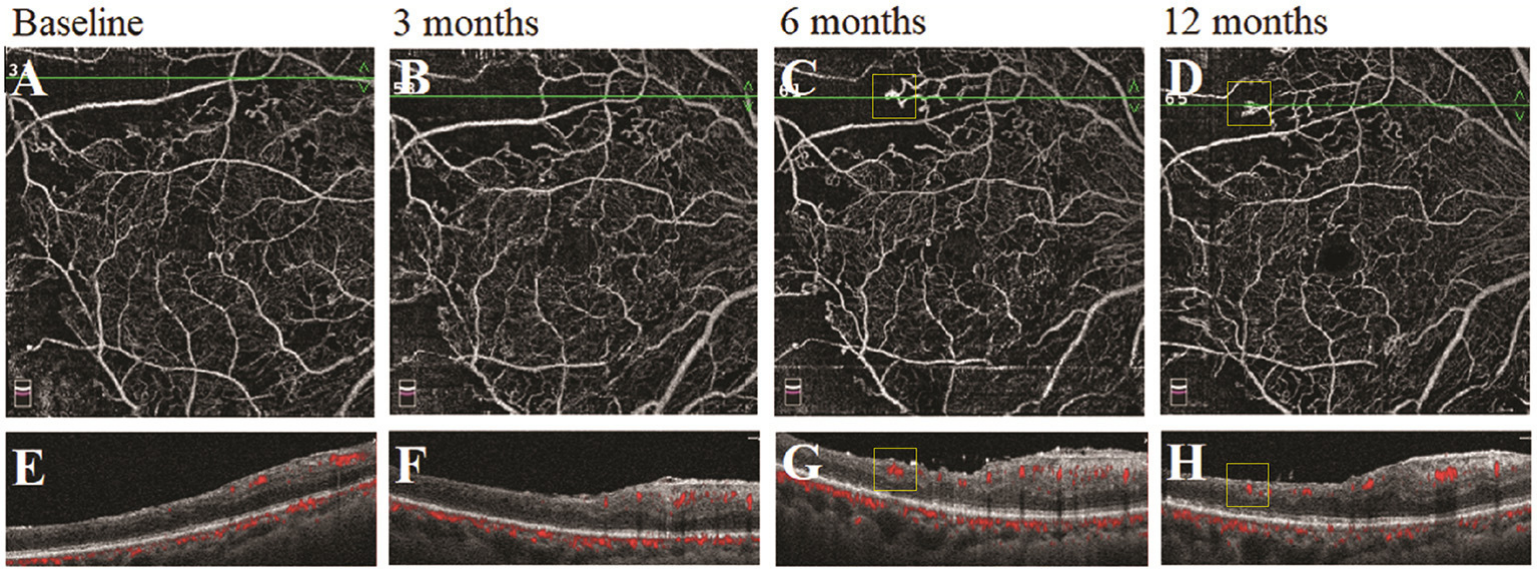
A typical severe nonproliferative diabetic retinopathy (NPDR) case showed the morphological changes of the similar intraretinal microvascular abnormalities (IRMAs) in the nonperfusion area following panretinal photocoagulation (PRP). **(A–D)** Retina en-face slab images of optical coherence tomography angiography (OCTA). **(E–H)** B-scan images of OCTA.

We observed the changes in the morphology of the neovascularization of the disk (NVD), from large and dense to small and loose, in eight eyes out of 12 eyes (67%) during the follow-up visit after PRP by using OCTA. Correspondingly, reduced fluorescein leakage of the NVD was observed on FFA (Figure 3). Furthermore, regression of neovascularization after PRP was documented in 18 eyes (18/22 eyes, 82%; Figure 4) and among them, the complete regression was in two eyes and partial regression was in 16 eyes post-PRP. No significant longitudinal changes of parameters, including BCVA, retinal VD and thickness, FAZ, peripapillary VD and RNFL thickness, GCC thickness, FLV and GLV, and SFCT, were observed in the total 18 PDR eyes with regression of neovascularization after PRP during the follow-up period (Supplementary Table 2). In addition, the new blood vessels, similar to the intraretinal microvascular abnormalities (IRMAs), were observed in the macular nonperfusion area of the severe NPDR eyes (Figure 5) and the changes of vascular morphology in the macular arch were also observed during the follow-up period.

## Discussion

This study was a prospective longitudinal study to investigate the long-term comprehensive changes of the retinal microvasculature, neural retina, and choroid following PRP. In this study, results showed no significant microvascular (macular and peripapillary VD); neural (whole macular thickness, peripapillary RNFL thickness, and GCC thickness); and the SFCT changes at 12 months after PRP, despite the increase of whole macular thickness, peripapillary RNFL, and GCC thickness at the early post-PRP phase. The regression of neovascularization and the unchanged BCVA in this study confirmed that PRP effectively alleviated retinal ischemia and protected the neural retina. These findings suggest that PRP may prevent diabetic microvascular, neural, and choroidal damage at 12 months in the treatment-naive patients with severe NPDR and PDR without DME.

There were no statistical differences in macular VD after PRP, although the decreased trend was observed at the 3–6 months follow-up, which was similar to the results reported in other literature (17–19). Microvascular abnormalities and subsequent capillary occlusion have been demonstrated to aggravate the decrease of the VD and enlargement of the FAZ with the progression of DR (11). However, Fawzi et al. found an overall increase in the flow metrics of all the macular capillary layers following PRP by the mathematical model, although no significant difference for the vascular density parameters was shown (17). Therefore, the unchanged macular VD and FAZ area during the 12 months following PRP in this study may indicate stabilization of the macular microcirculation and control of ischemia.

Although several studies showed a significant reduction in the macular choroidal thickness after PRP (20–22), results showed no significant change during the 12-month follow-up. This discrepancy might be due to the differences in the profiles of the patient. This study included the severe NPDR or PDR patients without DME, while the previous reports included the eyes with DME or with IVI. On one hand, the subfoveal choroid is thicker in the eyes with DME compared to those without the eyes with DME (22), which leads to a higher baseline level. On the other hand, the downregulation of VEGF due to the IVI may induce a decrease in choroidal blood flow and thickness (23), which perhaps affect the assessment of the macular choroidal thickness after PRP. Taken together, our results showing the long-term stability of VD in the macula, peripapillary area, and subfoveal choroid indicate an overall redistribution of the capillary blood flow to the posterior pole following PRP.

Although the VD in the posterior pole remained unchanged, macular retinal thickness significantly increased after PRP, especially at the fovea 1–6 months postoperatively. This finding may be explained by the retinal inflammation and edema caused by PRP in the early postoperative phase (24). However, we found that macular thickness was significantly decreased in the eyes receiving IVI plus PRP (Supplementary Table 3). This group included 10 eyes with CSME (central subfield macular thickness ≥ 300 μm) in seven patients with severe NPDR and PDR. All of the participants received one-time IVI before initial PRP within 7 days. Reinjection was performed if obvious CSME reoccurred during the 12-month follow-up. These findings supported by the other studies (25, 26) suggest that treatment with adjunctive IVI before or after PRP could reduce macular edema and stabilizes the blood-retinal barrier. Of course, it is necessary to perform a further study in a larger sample to confirm this finding.

The changes in the RNFL and GCC thickness showed similar trends that were significantly increased at 1 month following PRP, while the GLV and FLV were significantly decreased during the early post-PRP period. Over the long-term follow-up, they gradually returned to the baseline level. Many of the previous studies using OCT obtained similar results (27, 28). They considered that the retinal inflammation and edema caused by PRP at the early postoperative phase could damage the retinal neural cell and tissue and later recovery might be attributed to the absorption and healing of the edema and the laser-induced photoreceptors and ganglion cell damage. However, other studies showed PRP to decrease the thickness of the peripapillary RNFL and GCC (29) suggesting that the presence of diabetes itself could cause neurodegenerative changes in the retina and subsequent degeneration of RNFL by retinal cell loss in the late PRP phase. To the best of our knowledge, there are no relevant studies observing the changes of the RNFL and GCC after PRP using OCTA.

This study comprehensively evaluated the retinal microvascular, neural, and choroidal changes after PRP in the treatment-naive patients with severe NPDR and PDR during a long-term follow-up period. To achieve the standard differences among the visits, all the OCTA and OCT metrics were adjusted for age, sex, and AL. This study had some limitations. First, the limited field view provided by OCTA only showed the macular area without the laser spots and could not completely evaluate the whole retina. Thus, wide-field OCTA will improve the accuracy of evaluation in the future. Second, the patients were excluded due to the changes in treatment (such as IVI and PPV), which resulted in the absence of observation in some of the patients with DME or with aggravation. Third, the dropout of the subjects during the follow-up period, partially due to the COVID-19, might make the resulting bias. In addition, since PRP is generally recommended for the treatment of the severe stages of DR (severe NPDR and PDR), it is difficult to longitudinally observe the changing features of these parameters in the untreated patients with severe DR, which is the main cause for the lack of a control group in this study.

In conclusion, the unchanged BCVA, VD, the thickness of RNFL and GCC, and SFCT during the 12-month follow-up period suggest that PRP may prevent the retinal microvascular, neural, and choroidal damages in the eyes with the severe NPDR and PDR without DME. Inclusion of the other metrics of visual function such as contrast sensitivity and visual field testing will help to better understand the mechanism of PRP.

## Data Availability Statement

The original contributions presented in the study are included in the article/Supplementary Material, further inquiries can be directed to the corresponding authors.

## Ethics Statement

The studies involving human participants were reviewed and approved by Ethics Committee of Guangdong Provincial People's Hospital. The patients/participants provided their written informed consent to participate in this study.

## Author Contributions

TH and XL collected data, performed analyses, and wrote the manuscript. JX collected data and revised the manuscript. LZ provided patients' information and supervised the process. GZ interpreted data. AZ and XC collected data. YC and QM interpreted data, revised the manuscript, gained the fund, and supervised the process. All authors contributed to the article and approved the submitted version.

## Funding

This study was supported by the Guangdong Basic and Applied Basic Research Foundation (2019A1515010697, 2021A1515010921); the Guangzhou Science and Technology Program Project (202002030400); the National Natural Science Foundation, Beijing, China (82000897), and the Bethune·Lumitin Research Funding for the Young and Middle-aged Ophthalmologists (BJ-LM2019004J). The funding organizations had no role in the design or conduct of this research.

## Conflict of Interest

The authors declare that the research was conducted in the absence of any commercial or financial relationships that could be construed as a potential conflict of interest. The handling editor and the reviewer WC declared a shared affiliation with one of the authors XL at time of review.

## Publisher's Note

All claims expressed in this article are solely those of the authors and do not necessarily represent those of their affiliated organizations, or those of the publisher, the editors and the reviewers. Any product that may be evaluated in this article, or claim that may be made by its manufacturer, is not guaranteed or endorsed by the publisher.
