# Supplementary material for: Long-Term Retinal Neurovascular and Choroidal Changes After Panretinal Photocoagulation in Diabetic Retinopathy
Source: Front Med (Lausanne). 2021 Oct 18;8:752538. doi: 10.3389/fmed.2021.752538 (PMC8558304; doi:10.3389/fmed.2021.752538)
Supplement: Supplementary file 1 [file Table_1.DOCX]

Supplementary Table 1. Longitudinal Changes of Macular Thickness, Peripapillary Vessel Density, Retinal Nerve Fiber Layer Thickness and Ganglion Cell Complex after Panretinal Photocoagulation [mean (95% confidence interval)].

| Variables | Baseline | 1 month | 3-6 months | 12 months | *P*-value |
| --- | --- | --- | --- | --- | --- |
| Macular thickness (μm) |  |  |  |  |  |
| Whole | 309.09 (287.31-330.87) | 319.53 (297.33-341.73) ** | 320.07 (297.71-342.44) * | 311.27 (288.77-333.77) | **0.010** |
| Foveal | 267.15 (239.05-295.25) | 292.87 (263.67-322.08) *** | 286.86 (257.23-316.49) * | 289.16 (259.17-319.14) ** | **0.001** |
| Parafoveal | 335.59 (314.85-356.34) | 345.43 (324.14-366.73) * | 351.25 (329.74-372.77) ** | 343.34 (321.64-365.03) | **0.008** |
| Perifoveal | 312.2 (288.33-336.06) | 322.01 (297.65-346.36) * | 323.05 (298.51-347.59) * | 312.64 (287.93-337.34) | **0.031** |
| RNFL thickness (μm) |  |  |  |  |  |
| peripapillary | 122.37 (113.46-131.29) | 130.10 (120.89-139.31) *** | 125.47 (116.2-134.74) | 124.95 (115.59-134.31) | **0.001** |
| Superior Nasal | 142.07 (124.77-159.38) | 153.76 (135.39-172.14) * | 140.78 (122.09-159.47) | 148.64 (129.73-167.55) | 0.106 |
| Nasal Superior | 99.44 (91.27-107.60) | 104.18 (95.65-112.70) * | 103.96 (95.45-112.48) | 98.87 (90.21-107.54) | **0.022** |
| Nasal Inferior | 87.01 (79.74-94.28) | 94.40 (86.67-102.13) ** | 89.98 (82.15-97.81) | 92.63 (84.67-100.6)* | **0.006** |
| Inferior Nasal | 146.23 (132.41-160.04) | 152.30 (138.18-166.42) * | 145.28 (131.1-159.46) | 143.46 (129.19-157.73) | **0.024** |
| Inferior Temporal | 164.84 (150.79-178.89) | 171.95 (157.5-186.41) * | 170.86 (156.32-185.4) | 163.99 (149.34-178.65) | **0.022** |
| Temporal Inferior | 97.53 (89.04-106.02) | 101.87 (92.69-111.04) | 103.40 (94.10-112.70) | 95.28 (85.79-104.77) | 0.082 |
| Temporal Superior | 103.28 (92.01-114.56) | 109.47 (97.71-121.24) * | 109.39 (97.53-121.26) | 107.63 (95.62-119.63) | 0.082 |
| Superior Temporal | 157.06 (142.7-171.43) | 167.28 (153.06-181.5) ** | 164.22 (149.86-178.58) | 161.91 (147.35-176.46) | **＜0.001** |
| GCC |  |  |  |  |  |
| GCC thickness (μm) | 113.83 (104.45-123.20) | 121.49 (111.68-131.29) ** | 116.93 (106.91-126.95) | 117.27 (107.22-127.32) | **0.039** |
| GLV (%) | 1.93 (1.19-2.67) | 0.75 (0.04-1.51) ** | 1.33 (0.53-2.14) | 2.10 (1.31-2.89) | **0.006** |
| FLV (%) | 1.55 (0.81-2.29) | 0.73 (0.05-1.55) * | 1.11 (0.31-1.91) | 1.87 (1.08-2.66) | **0.007** |

VD = vessel density; RNFL = retinal nerve fiber layer; GCC = ganglion cell complex; GLV = global loss volume; FLV = focal loss volume. *P*-value calculated using linear mixed-effects model before and after panretinal photocoagulation adjusting for age, sex and axial length. ^*^*P* < 0.05, ^**^*P* < 0.01, ^***^*P* < 0.001 *vs.* baseline.
