# Supplementary material for: Long-Term Retinal Neurovascular and Choroidal Changes After Panretinal Photocoagulation in Diabetic Retinopathy
Source: Front Med (Lausanne). 2021 Oct 18;8:752538. doi: 10.3389/fmed.2021.752538 (PMC8558304; doi:10.3389/fmed.2021.752538)
Supplement: Supplementary file 2 [file Table_2.DOCX]

Supplementary Table 2. Longitudinal Best-corrected Visual Acuity, Microvascular, Neural and Choroidal Changes in PDR eyes with Regression of Neovascularization after Panretinal Photocoagulation [mean (95% confidence interval)].

| Variables | Baseline | 1 month | 3-6 months | 12 months | *P*-value |
| --- | --- | --- | --- | --- | --- |
| BCVA (LogMAR) | 0.28 (0.08-0.49) | 0.14 (0.02-0.34) | 0.20 (0.05-0.41) | 0.18 (0.04-0.37) | 0.272 |
| Macular SCP VD (%) |  |  |  |  |  |
| Whole | 48.84 (43.86-53.83) | 49.03 (43.65-54.87) | 47.58 (42.64-52.56) | 48.55 (43.29-53.67) | 0.532 |
| Foveal | 8.57 (-3.21-20.34) | 12.39 (-4.90-29.67) ^*^ | 11.66 (-0.16-23.47) | 10.05 (-1.74-21.84) | 0.414 |
| Parafoveal | 49.83 (44.29-55.36) | 48.74 (42.60-55.42) | 48.60 (43.17-54.09) | 48.19 (42.29-53.96) | 0.635 |
| Perifoveal | 50.00 (44.55-55.45) | 49.91 (44.29-56.52) | 48.95 (43.52-54.38) | 49.74 (43.96-55.37) | 0.519 |
| Macular DCP VD (%) |  |  |  |  |  |
| Whole | 45.23 (40.07-50.39) | 45.49 (38.45-51.71) | 41.80 (36.64-46.99) | 42.70 (37.07-48.5) | 0.673 |
| Foveal | 16.16 (4.37-27.94) | 18.67 (5.15-32.19) | 17.78 (6.86-28.69) | 12.39 (1.28-23.50) | 0.583 |
| Parafoveal | 49.34 (43.45-55.23) | 49.11 (41.31-56.91) | 44.63 (37.79-51.48) | 47.35 (40.08-54.61) | 0.724 |
| Perifoveal | 47.37 (42.12-52.63) | 47.08 (40.10-53.52) | 43.86 (38.62-49.14) | 45.11 (39.39-50.98) | 0.805 |
| Macular thickness (μm) |  |  |  |  |  |
| Whole | 278.44 (214.30-342.58) | 280.13 (212.97-347.30) | 285.79 (221.65-349.93) | 264.40 (199.21-329.61) | 0.566 |
| Foveal | 254.55 (200.44-308.66) | 257.12 (191.08-323.17) | 263.00 (208.89-317.11) | 256.67 (198.10-315.24) | 0.687 |
| Parafoveal | 319.93 (276.95-362.91) | 322.03 (273.77-370.30) | 330.73 (287.75-373.71) | 305.35 (260.49-350.22) | 0.497 |
| Perifoveal | 276.13 (202.61-349.66) | 278.03 (200.98-355.09) | 284.78 (211.26-358.31) | 259.49 (184.73-334.25) | 0.555 |
| FAZ (6.0 mm scan) |  |  |  |  |  |
| FAZ (mm2) | 0.54 (0.23-0.85) | 0.46 (0.07-0.87) | 0.46 (0.16-0.76) | 0.54 (0.20-0.88) | 0.813 |
| Perimeter (mm) | 3.05 (1.98-4.12) | 2.73 (1.37-4.18) | 2.68 (1.63-3.75) | 2.89 (1.69-4.09) | 0.875 |
| AI | 1.15 (1.05-1.25) | 1.12 (0.99-1.26) | 1.09 (0.99-1.19) | 1.09 (0.98-1.20) | 0.884 |
| FD-300 | 50.26 (44.39-56.12) | 48.14 (42.26-56.98) | 44.35 (38.63-50.19) | 48.13 (41.72-54.59) | 0.176 |
| Peripapillary VD (%) |  |  |  |  |  |
| Peripapillary | 51.8 (48.22-55.42) | 49.04 (44.57-53.52) | 51.61 (48.01-55.21) | 53.27 (49.34-57.20) | 0.061 |
| RNFL thickness (μm) |  |  |  |  |  |
| peripapillary | 141.49 (117.19-165.79) | 149.40 (123.37-175.44) | 150.78 (126.49-175.08) | 138.62 (113.71-163.53) | 0.437 |
| GCC |  |  |  |  |  |
| GCC thickness (μm) | 124.33 (96.83-151.82) | 126.24 (96.73-156.72) | 129.76 (102.15-157.25) | 119.33 (87.57-144.28) | 0.267 |
| GLV (%) | 1.67 (0.81-2.52) | 0.50 (-0.24-0.69) ^*^ | 1.44 (0.63-2.27) | 2.56 (1.54-3.61) | **0.025** |
| FLV (%) | 1.65 (0.82-2.51) | 0.20 ( -0.53-1.27) ^*^ | 1.21 (0.42-2.11) | 2.33 (1.30-3.56) | **0.034** |
| SFCT (μm) | 244.22 (183.85-304.59) | 260.77 (164.66-347.35) | 264.35 (174.32-336.20) | 281.64 180.14-367.33) | 0.302 |

BCVA (LogMAR) = best corrected visual acuity (logarithm of the minimum angle of resolution); SCP = superficial capillary plexus; DCP = deep capillary plexus; VD = vessel density; FAZ = foveal avascular zone; AI = acircularity index; RNFL = retinal nerve fiber layer; GCC = ganglion cell complex; GLV = global loss volume; FLV = focal loss volume; SFCT = subfoveal choroidal thickness. *P*-value calculated using linear mixed-effects model before and after panretinal photocoagulation adjusting for age, sex and axial length. ^*^*P* < 0.05 *vs.* baseline.
