# Supplementary material for: Long-Term Retinal Neurovascular and Choroidal Changes After Panretinal Photocoagulation in Diabetic Retinopathy
Source: Front Med (Lausanne). 2021 Oct 18;8:752538. doi: 10.3389/fmed.2021.752538 (PMC8558304; doi:10.3389/fmed.2021.752538)
Supplement: Supplementary file 3 [file Table_3.DOCX]

Supplementary Table 3. Longitudinal Best-corrected Visual Acuity, Microvascular, Neural and Choroidal Changes after Intravitreal Anti-Vascular Endothelial Growth Factor Injection plus Panretinal Photocoagulation [mean (95% confidence interval)].

| Variables | Baseline | 1 month | 3-6 months | 12 months | *P*-value |
| --- | --- | --- | --- | --- | --- |
| BCVA (Log MAR) | 0.63 (0.19-1.08) | 0.46 (0.02-0.91) | 0.45 (0.01-0.89) * | 0.52 (0.07-0.97) | 0.135 |
| Macular SCP VD (%) |  |  |  |  |  |
| Whole | 42.84 (39.00-46.69) | 40.8 (36.76-44.84) | 38.88 (35.20-42.56) * | 39.88 (35.79-43.97) | 0.111 |
| Foveal | 25.43 (17.03-33.83) | 20.4 (11.60-29.19) | 21.26 (13.20-29.32) | 21.63 (12.73-30.53) | 0.485 |
| Parafoveal | 41.39 (36.61-46.17) | 40.64 (35.67-45.60) | 38.09 (33.47-42.71) | 39.5 (34.49-44.51) | 0.297 |
| Perifoveal | 43.41 (39.38-47.44) | 41.25 (37.06-45.43) | 39.50 (35.60-43.40) * | 40.32 (36.10-44.54) | 0.088 |
| Macular DCP VD (%) |  |  |  |  |  |
| Whole | 43.95 (40.18-47.71) | 41.20 (37.18-45.22) | 40.47 (36.93-44.01) | 40.07 (35.95-44.18) | 0.193 |
| Foveal | 35.93 (28.31-43.56) | 31.58 (23.63-39.53) | 34.15 (26.81-41.5) | 34.16 (26.13-42.19) | 0.582 |
| Parafoveal | 48.41 (44.05-52.78) | 46.50 (41.87-51.13) | 46.24 (42.10-50.37) | 44.98 (40.26-49.69) | 0.492 |
| Perifoveal | 44.56 (40.47-48.64) | 41.20 (36.83-45.57) | 40.69 (36.86-44.51) | 40.40 (35.92-44.88) | 0.185 |
| Macular thickness (μm) |  |  |  |  |  |
| Whole | 391.12 (332.28-449.96) | 346.27 (284.88-407.66) | 376.31 (319.67-432.95) | 363.71 (301.68-425.74) | 0.325 |
| Foveal | 442.86 (319.44-566.27) | 317.54 (186.15-448.93) * | 399.93 (283.55-516.30) | 395.36 (261.16-529.55) | 0.261 |
| Parafoveal | 443.09 (356.35-529.82) | 375.30 (282.78-467.83) | 421.40 (339.78-503.02) | 409.32 (314.69-503.94) | 0.488 |
| Perifoveal | 385.45 (321.35-449.55) | 345.70 (279.18-412.23) | 374.25 (312.20-436.29) | 361.09 (294.02-428.16) | 0.446 |
| FAZ (6.0 mm scan) |  |  |  |  |  |
| FAZ (mm^2^) | 0.28 (0.13-0.42) | 0.28 (0.14-0.43) | 0.29 (0.14-0.43) | 0.26 (0.11-0.4) | 0.920 |
| Perimeter (mm) | 2.13 (1.53-2.73) | 2.08 (1.46-2.69) | 2.09 (1.50-2.69) | 2.10 (1.49-2.71) | 0.986 |
| AI | 1.17 (1.08-1.26) | 1.10 (1.01-1.20) | 1.12 (1.04-1.21) | 1.16 (1.06-1.26) | 0.371 |
| FD-300 | 47.28 (42.34-52.22) | 46.33 (41.16-51.51) | 46.74 (41.99-51.48) | 44.72 (39.49-49.95) | 0.762 |
| Peripapillary VD (%) |  |  |  |  |  |
| Peripapillary | 50.08 (43.08-57.08) | 49.06 (42.05-56.07) | 48.88 (41.87-55.88) | 48.7 (41.69-55.72) | 0.637 |
| RNFL thickness (μm) |  |  |  |  |  |
| peripapillary | 130.48 (111.05-149.92) | 136.93 (117.45-156.41) * | 131.50 (112.05-150.94) | 126.13 (106.65-145.61) | 0.057 |
| GCC |  |  |  |  |  |
| GCC thickness (μm) | 158.79 (120.51-197.07) | 159.30 (119.75-198.85) | 151.82 (114.18-189.46) | 155.45 (116.93-193.97) | 0.863 |
| GLV (%) | 0.98 (-0.68-2.64) | 0.28 (-1.59-2.14) | 0.10 (-1.37-1.16) | 0.15 (-1.54-1.84) | 0.547 |
| FLV (%) | 0.96 (-0.63-2.56) | 0.28 (-1.51-2.08) | 0.08 (-1.33-1.17) | 0.16 (-1.47-1.79) | 0.540 |
| SFCT (μm) | 278.22 (244.93-311.52) | 288.17 (251.82-324.51) | 260.92 (227.63-294.22) | 257.22 (222.99-291.46) | 0.105 |

BCVA = best-corrected visual acuity; SCP = superficial capillary plexus; DCP = deep capillary plexus; VD = vessel density; FAZ = foveal avascular zone; AI = acircularity index; RNFL = retinal nerve fiber layer; GCC = ganglion cell complex; GLV = global loss volume; FLV = focal loss volume; SFCT = subfoveal choroidal thickness. *P*-value calculated using linear mixed-effects model before and after panretinal photocoagulation adjusting for age, sex and axial length. ^*^*P* < 0.05 *vs.* baseline.
