# Supplementary material for: Long-Term Retinal Neurovascular and Choroidal Changes After Panretinal Photocoagulation in Diabetic Retinopathy
Source: Front Med (Lausanne). 2021 Oct 18;8:752538. doi: 10.3389/fmed.2021.752538 (PMC8558304; doi:10.3389/fmed.2021.752538)
Supplement: Supplementary file 4 [file Image_1.PDF]

Supplementary figure

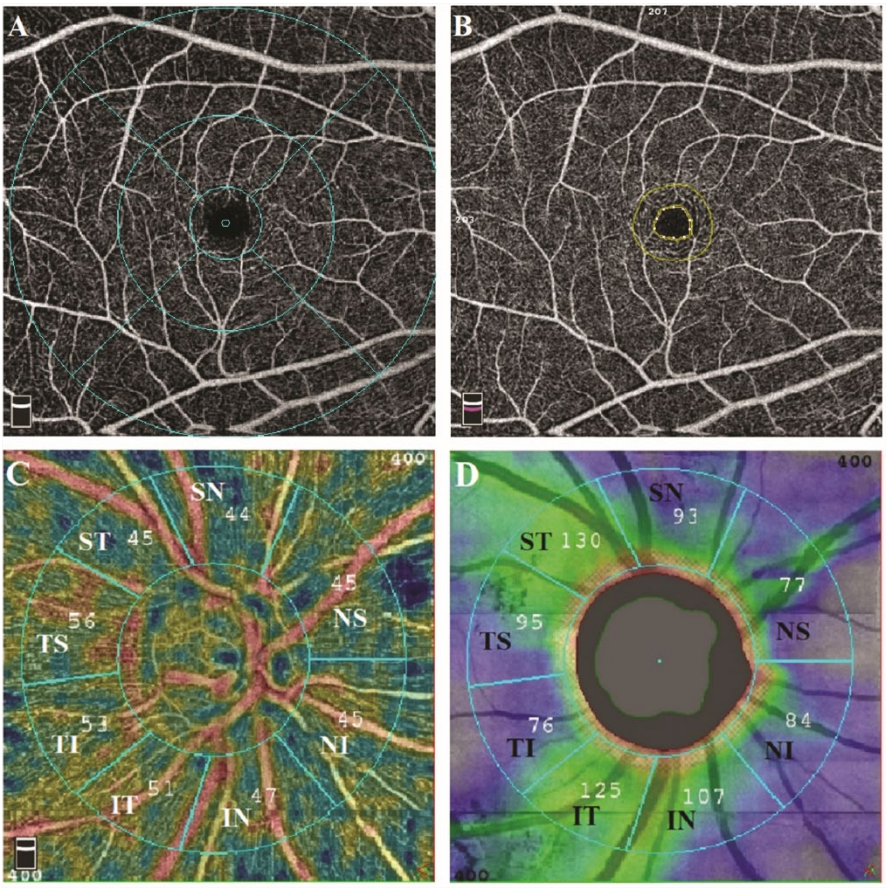

Supplementary figure 1. Angio retina and disc measurement zones. (A) A 6.0 mm scan centered on the fovea at the superficial capillary plexus (SCP). The Early Treatment Diabetic Retinopathy Study grid in the macular area comprises three concentric rings: 1 mm center (fovea), 1 to 3 mm (parafovea), and outer ring of 3 to 6 mm diameters (perifovea). (B) A foveal avascular zone (FAZ) image of 6.0 mm scan. (C) Peripapillary VD. (D) Peripapillary retinal thickness. Peripapillary region at 4.5 mm scan is defined by two rings of 2 mm and 4 mm centered on the disc center. The modified eight peripapillary sectors grid aims to follow retinal nerve fiber layer (RNFL) distribution and sectored to provide easier correlation with the visual field. The eight peripapillary sectors include nasal superior (NS), nasal inferior (NI), inferior nasal (IN), inferior temporal (IT), temporal inferior (TI), temporal superior (TS), superior temporal (ST), and superior nasal (SN).
